# Silencing VDAC1 to Treat Mesothelioma Cancer: Tumor Reprograming and Altering Tumor Hallmarks

**DOI:** 10.3390/biom12070895

**Published:** 2022-06-27

**Authors:** Swaroop Kumar Pandey, Renen Machlof-Cohen, Manikandan Santhanam, Anna Shteinfer-Kuzmine, Varda Shoshan-Barmatz

**Affiliations:** 1 Department of Life Sciences, Ben-Gurion University of the Negev, Beer-Sheva 84105, Israel; pandey@post.bgu.ac.il (S.K.P.); renenmac@post.bgu.ac.il (R.M.-C.); santhana@post.bgu.ac.il (M.S.); 2 The National Institute for Biotechnology in the Negev, Ben-Gurion University of the Negev, Beer-Sheva 84105, Israel; shteinfe@post.bgu.ac.il

**Keywords:** mesothelioma, metabolism, mitochondria, VDAC1

## Abstract

Mesothelioma, an aggressive cancer with a poor prognosis, is linked to asbestos exposure. However, carbon nanotubes found in materials we are exposed to daily can cause mesothelioma cancer. Cancer cells reprogram their metabolism to support increased biosynthetic and energy demands required for their growth and motility. Here, we examined the effects of silencing the expression of the voltage-dependent anion channel 1 (VDAC1), controlling the metabolic and energetic crosstalk between mitochondria and the rest of the cell. We demonstrate that VDAC1 is overexpressed in mesothelioma patients; its levels increase with disease stage and are associated with low survival rates. Silencing VDAC1 expression using a specific siRNA identifying both mouse and human VDAC1 (si-m/hVDAC1-B) inhibits cell proliferation of mesothelioma cancer cells. Treatment of xenografts of human-derived H226 cells or mouse-derived AB1 cells with si-m/hVDAC1-B inhibited tumor growth and caused metabolism reprogramming, as reflected in the decreased expression of metabolism-related proteins, including glycolytic and tricarboxylic acid (-)cycle enzymes and the ATP-synthesizing enzyme. In addition, tumors depleted of VDAC1 showed altered microenvironments and inflammation, both associated with cancer progression. Finally, tumor VDAC1 silencing also eliminated cancer stem cells and induced cell differentiation to normal-like cells. The results show that silencing VDAC1 expression leads to reprogrammed metabolism and to multiple effects from tumor growth inhibition to modulation of the tumor microenvironment and inflammation, inducing differentiation of malignant cells. Thus, silencing VDAC1 is a potential therapeutic approach to treating mesothelioma.

## 1. Introduction

Mesothelioma is an aggressive form of cancer that arises from the mesothelium that lines the pleura and other serous cavities (such as the peritoneum, pericardium, and tunica vaginalis testis). There are three primary types of mesotheliomas: pleural mesothelioma (lungs, 75–80% of all cases), peritoneal mesothelioma (abdomen, 20–25% of all cases), and pericardial mesothelioma (heart, less than 1% of all cases [1,2].

The most common cause of human malignant mesothelioma (more than 80% of the cases) is exposure to pollutants, particularly asbestos. Asbestos, principally composed of two types of fibers, serpentine and amphibole, was largely used until the 1980s and 1990s. As inhalation of asbestos is a risk factor for the development of mesothelioma, asbestos is now banned in at least 60 countries worldwide. Even so, according to the World Health Organization (WHO), 125 million workers continue to be exposed to asbestos on a daily basis, and it is estimated that 107,000 deaths still occur annually [3].

Diseases caused by asbestos develop slowly, and with time the retention of asbestos fibers in the pleural cavity is crucial for disease development, and chronic inflammation plays an important role in carcinogenesis [4]. The latency period of this disease can last up to 40 years [5]. Median survival of patients with mesothelioma from time of diagnosis ranges between 8 to 14 months and there are few biomarkers and no cure available [6].

Other known risk factors for mesothelioma include therapeutic radiation [7] and genetic factors [8]. Recently, exposure to carbon nanotubes (CNTs), which are used to produce televisions), tennis rackets, sports cars, and computer motherboards, was also reported as a potential cause of mesothelioma [9,10]. Several thousand tons of CNTs are produced each year due to their high electrical conductivity, with their thermal conductivity ten times that of copper and tensile strength one hundred times that of steel [11]. In vivo studies have indicated that CNTs can cause sustained inflammation and fibrosis of the pleura and can also induce tumor development in genetically susceptible or peritoneally exposed rodent models [12]. Data comparing prolonged pleural exposure of mice to occupationally relevant doses of CNTs or asbestos are lacking, and the molecular mechanisms underlying fiber-induced carcinogenesis have not yet been explored [13,14].

Genetic analyses have identified several key genetic alterations in end-stage disease, with the most common deletions or mutations being inactivation of the *CDKN2A* locus that encodes for the cell cycle inhibitor proteins (p16INK4A, p15INK4B, and p14ARF) in tumor suppressor neurofibromatosis Type 2 (NF2), and BAP1, encoding a nuclear deubiquitinase [15]. It was suggested that two main pathways—p53/DNA repair and PI3K-AKT—are associated with mesothelioma progression [16].

Mesothelin (MSLN), a 40-kDa glycoprotein found in normal mesothelial cells [17], seems to be overexpressed in about 30% of all cancers [18], including in mesothelioma, ovarian cancer, pancreatic cancer, and other solid tumors [19]. In normal cells, MSLN is proposed to be involved in cell adhesion, differentiation, and signal transduction. In cancerous cells, it may be involved in the promotion of proliferation, cell migration and spread, chemotherapy resistance, and inhibition of apoptosis [20].

The overexpression of MSLN activates multiple intracellular pathways, including nuclear factor-kappaB (NF-κB), mitogen-activated protein kinase (MAPK), and phosphoinositide 3-kinases (PI3K) pathways, with the consequent promotion of cell proliferation, migration, and metastasis to distal sites and the inhibition of apoptosis [21,22]. Asbestos is proposed to induce oncogenesis via the activation of the NF-κB-dependent pathway [23]. 

Although different types of treatments are available for patients with malignant mesothelioma, there is yet no cure, and the need for new targets and treatments is urgently needed. Surgery is applied with a prognostic/palliative intent, while standard chemotherapy is based on treatment with cisplatin combined with pemetrexed or raltitrexed. New therapeutic approaches under evaluation include inhibitors such as angiogenesis [24], mTOR [25], and histone deacetylase [26]. 

Given the overexpression of MSLN in over 80% of total malignant mesothelioma cases, new agents targeting MSLN are under evaluation in clinical trials and are considered a very promising therapy strategy. Indeed, amatuximab, a chimeric monoclonal anti-MSLN antibody, showed an enhanced anti-tumor effect of gemcitabine against mesothelin, highly expressing pancreatic cancer cell metastasis in a mouse model [27]. For malignant pleural mesothelioma, the FDA has approved bevacizumab with treatments such as cisplatinum/pemetrexed [28]. It was recently shown that treatment with anti-PD-L1 resulted in a beneficial response of nearly 12 months [29]. However, the reported disadvantages of this therapy are its low (10–20%) positive response in patients and a decrease of only about 50–60% of the disease state [19].

One of the proteins controlling cell energy and metabolic homeostasis and apoptosis [30,31] that is highly expressed in various cancer cell lines and different tumors [31,32,33,34,35] is a mitochondrial protein, namely, voltage-dependent anion channel 1 (VDAC1). VDAC1 at the outer mitochondrial membrane (OMM) mediates the metabolic and Ca^2+^ exchange between the mitochondria and the rest of the cell, transporting metabolites, ions, nucleotides, pyruvate, malate, succinate, Ca^2+^, and more, thereby regulating mitochondrial activity. Thus, VDAC1 plays a central role in cell energy and metabolism homeostasis, mediating the metabolic crosstalk between the mitochondria and cytosol [30,31]. VDAC1 also plays a key role in apoptosis, participating in the release of apoptotic factors from the mitochondria and interacting with anti-apoptotic proteins, preventing their activity [30,31]. As a key regulator of metabolic and energy reprogramming, disrupting cancer energy and metabolism homeostasis by VDAC1 depletion in tumor cells is thus expected to affect cancer development and survival. Indeed, we have demonstrated that silencing VDAC1 expression using VDAC-specific siRNA reduced cellular ATP levels and cell proliferation, and in mouse models of glioblastoma, and cervical and lung cancers, it induced metabolic reprograming, inhibited tumor development and growth and angiogenesis, and altered the tumor microenvironment [32,36,37,38,39,40,41]. 

In this study, we used siRNA-mediated silencing of VDAC1 expression in mesothelioma cell lines of mouse and human origin and in mouse models using these same mesothelioma cell lines. Using human and mouse VDAC1 recognizing siRNA (si-m/hVDAC1-B), we found that VDAC1 depletion inhibited cell proliferation, induced metabolism reprograming, altered the tumor microenvironment, eliminated cancer stem cells, and induced cell differentiation. Thus, VDAC1 silencing can serve as a novel therapeutic approach to treat mesothelioma.

## 2. Materials and Methods

### 2.1. Materials

The cell transfection agents siLenFect was obtained from Bio-Rad (Hercules, CA, US) and JetPEI from PolyPlus (Illkirch, France). 2′-*O*-methyl-modified h/mVDAC1-B-siRNA and non-targeting (NT)-siRNA were obtained from Genepharma (Suzhou, China). Matrigel matrix was obtained from Corning (Oneonta, NY, USA)). Trypan blue, Triton X-100, Tween-20, hematoxylin, eosin, 4′,6-diamidino-2-phenylindole (DAPI), and dimethyl sulfoxide (DMSO) were obtained from Sigma-Aldrich (St. Louis, MO, USA). Dulbecco’s modified Eagle’s medium (DMEM), Roswell Park Memorial Institute media, and phosphate buffered saline (PBS) were obtained from Gibco-Thermo Fisher Scientific (Waltham, MA, USA). Normal goat serum (NGS), Hank’s balanced salts solution (HBSS), Waymouth MB 752/1 medium, DEPC-treated water, fetal bovine serum (FBS), trypsin, EDTA, a chemiluminescence detection kit for HRP (EZ-ECL), and penicillin and streptomycin were obtained from Biological Industries (Beit Haemek, Israel). A protease inhibitor cocktail set III, EDTA-free, was purchased from Millipore (Burlington, MA, US). 3,3-Diaminobenzidine (DAB) was obtained from Vector laboratories (Newark, CA, USA). Primary and secondary antibodies, their source, and their dilutions are detailed in Table 1.

### 2.2. Biomax Tissue Array 

Mesothelioma and healthy tissue microarrays (catalog numbers MS801b and MS1001a) were purchased from Biomax US (US Biomax, Inc., MD, USA), containing normal (n = 8), benign (n = 2), and mesothelioma cancer tissues (n = 52).

### 2.3. Cell Culture

H226 (squamous cell carcinoma, human mesothelioma) and AB1 (mouse malignant mesothelioma) cell lines were grown in RPMI supplemented with 10% FBS, 100 U/mL penicillin, and 100 μg/mL streptomycin and maintained in a humidified atmosphere at 37 °C and 5% CO_2_. 

### 2.4. Si-RNA

Non-targeting (si-NT) and si-h/mVDAC1-B, both 2′-*O*-methyl-modified, were synthesized and obtained from Genepharma (Suzhou, China).

si-NT, sense: 5′GCAAACAUCCCAGAGGUAU3′,
Anti-sense: 5′AUACCUCUGGGAUGUUUGC3′
si-h/mVDAC1-B, sense: 5′GAAUAGCAGCCAAGUAUCAGtt 3′
Anti-sense: 5′CUGAUACUUGGCUGCUAUUCtt 3′
Nucleotides colored in red and underlined were 2′-*O*-methyl-modified.

### 2.5. Sulforhodamine B (SRB) Assay for Cell Proliferation

For the cell proliferation assay, 24 h post-cell transfection with si-NT or si-hVDAC1-B, cells were counted and seeded in 96-well plates. After an additional 24, 48, or 96 h, cells were washed with PBS, fixed with 10% trichloroacetic acid (TCA) for 1.5 h, and subsequently stained with SRB. SRB was extracted from the cells using 100 mM Tris-base, and absorbance at 510 nm was determined using an Infinite M1000 plate reader (Tecan, Männedorf, Switzerland).

### 2.6. Xenograft Mouse Model

Syngeneic model: mouse mesothelioma AB1 cells (5 × 10^5^ in 0.1 mL PBS) were sub-cutaneously (s.c.) inoculated in the hind leg flanks of 6–7-week-old BALB/c female mice. H226 human mesothelioma cells (5 × 10^6^ in 0.1 mL PBS with 20% Matrigel) were s.c. inoculated into 6–7-week-old athymic male nude-Foxn1nu mice. Every three days, tumors were measured with a digital caliper in two dimensions, and tumor volume was calculated as follows: volume = (X^2^ × Y)/2, where X and Y are the short and long tumor dimensions, respectively. The mice with xenografts reaching a volume of 60–110 mm^3^ were randomized sub-grouped (n = 6) and treated with si-NT or si-m/hVDAC1-B to a final concentration of 100 nM after mixing with in vivo JetPEI, a delivery reagent, according to the manufacturer’s instructions. Injection into the established s.c. tumors was carried out every three days.

At the experiment end point, the mice were sacrificed using CO_2_ gas, and tumors were excised, weighed, and fixed with formaldehyde. Experimental protocols were approved by the Institutional Animal Care and Use Committee of Ben-Gurion University.

### 2.7. Immunohistochemistry (IHC) and Immunofluorescence (IF) of Tumor Tissue Sections

Immunohistochemical staining was performed on 5 μm-thick formalin-fixed and paraffin-embedded tumor tissue sections and deparaffinized by placing the slides at 60 °C for 1 h using xylene. Subsequently, the tissue sections were re-dried with a graded ethanol series (100–50%). Antigen retrieval for certain proteins (HK-I, LDH, citrate synthase, and ATP synthesis 5A) was performed in a 0.01 M citrate buffer (pH 6.0). After rinsing sections in PBS containing 0.1% Triton-X100 (pH 7.4), non-specific antibody binding was reduced by incubating the sections with 10% NGS for 2 h. After removing excess serum, sections were incubated overnight at a temperature of 4 °C with primary antibodies (sources and dilutions used are listed in Table 1). Parts were washed with PBST. For IHC, endogenous peroxidase activity was blocked by incubating the sections with 3% H_2_O_2_ for 15 min. After thorough rinsing with PBST, the sections were incubated for 2 h with mouse or rabbit antibodies (1:250) and secondary antibodies coupled to HRP, as needed. Sections were washed five times with PBST, and the oxidase reaction was subsequently shown by incubation with 3,3-diaminebenzidine (DAB) (ImmPact-DAB, Newark, CA, USA). After rinsing with water, the sections were counterstained with hematoxylin, and mounted with Vectashield mounting medium (Vector Laboratories, Newark, CA, USA).

For quantification, sections were scanned with a panoramic scanner (panoramic MIDI II, 3DHISTH, Budapest, Hungary) and analyzed using the IHC Profiler plugin within ImageJ software [42]. Five images from each section of randomly selected areas were examined. The software measured the intensity of the brown color—a HRP reaction product. In order to enhance the accuracy of quantification, only the percentage of “high positive” staining intensity was measured [43].

Non-specific control experiments were performed using the same protocols but omitting incubation with the primary antibodies.

For immunofluorescence staining following section incubation with primary antibodies and washing with PBS, sections were incubated for 1 h with the appropriate secondary antibody (Table 1) and viewed with an Olympus IX81 confocal microscope. Quantification analysis of stained slides of IF was done using an ImageJ software.

### 2.8. Protein Extraction

For cell cultures, the cell pellet was incubated on ice for 10 min with 50–150 µL lysis buffer (50 mM Tris-HCl, pH 7.5; 150 mM NaCl; 1 mM EDTA; 1.5 mM MgCl_2_; 10% glycerol; 1% Triton-X100; supplemented with a protease inhibitor cocktail (Calbiochem, UK)). Lysate from the cells was centrifuged for 15 min at 20,000× *g* at 4 °C. The upper layer was collected into a new tube.

To extract proteins from tumors, tissues were solubilized in a lysis buffer (50 mM Tris-HCl, pH 7; 150 mM NaCl; 1 mM EDTA; 1.5 mM MgCl_2_; 10% glycerol and 1% Triton X-100; and a protease inhibitor cocktail (Calbiochem, UK)), followed by sonication and centrifugation (10 min, 600× *g*). The protein concentration of each lysate was determined using a Lowry assay. Samples were stored at −20 °C until analysis by gel electrophoresis and immunoblotting.

### 2.9. Gel Electrophoresis and Immunoblotting

Following SDS-PAGE, proteins were transferred to membranes containing proteins and were blocked with 5% non-fat dry milk and 0.1% Tween-20 in TBS, incubated with the primary antibodies and then with HRP-conjugated anti-mouse or anti-rabbit IgG (sources and dilutions as detailed in Table 1). Enhanced chemiluminescent substrate (Biological industries, Beit-Ha-Emek, Israel) was used for the detection of HRP activity.

### 2.10. Statistical Analysis

Data are shown as the mean ± SEM of at least three independent experiments unless specified differently. Significance of differences was calculated by a two-tailed Student’s *t*-test. Statistical significance was reported at *p* ≤ 0.05 (*), *p* ≤ 0.01 (**), *p* ≤ 0.001 (***), or *p* ≤ 0.0001 (***).

## 3. Results

### 3.1. High Expression of VDAC1 in Mesothelioma Is Associated with Low Survival Rate

**Figure 1:**
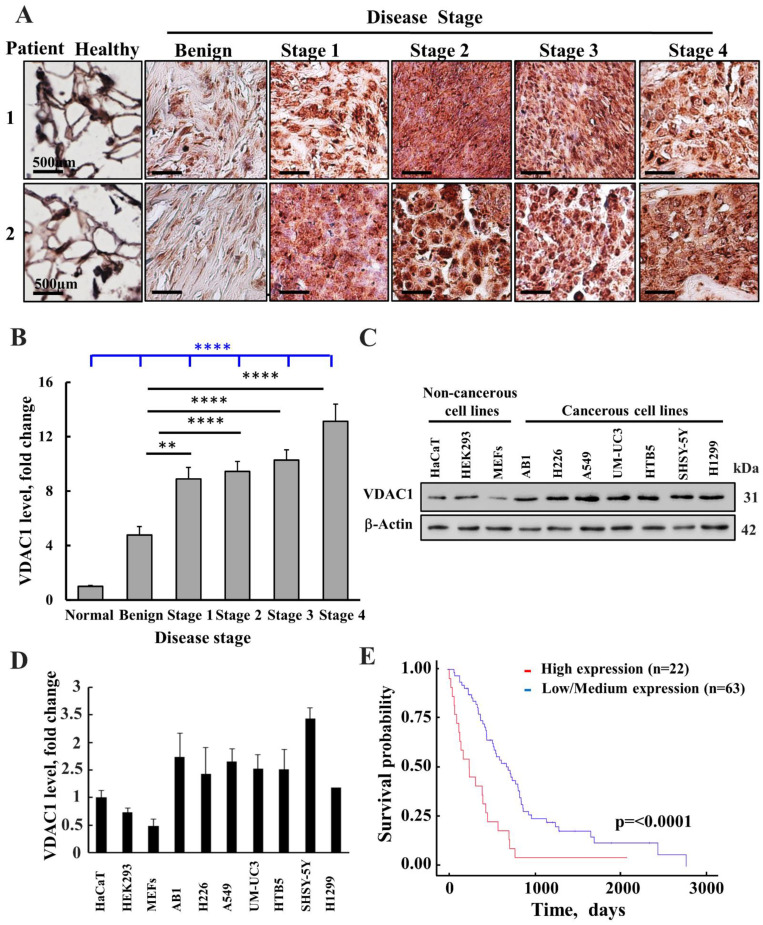
VDAC1 is over-expressed in mesothelioma patients and cancer cell lines. (**A**) Representative IHC staining for VDAC1 in healthy (n = 8) and mesothelioma at different disease stages (n = 52). Samples from tissue microarray slide (US Biomax MS801b; normal, benign and malignant; US Biomax MS1001a; malignant) samples from two patients at each disease stage are presented. (**B**) Quantitative analysis of VDAC1 levels in the tissue array samples at different disease stages. The results are means ± SEM (n = 18, 14, 14, and 6 for Stages 1, 2, 3, and 4, respectively), ** *p* ≤ 0.01; **** *p* ≤ 0.0001. Black lines are relative to benign tumors. The blue line is relative to healthy donors. (**C**) VDAC1 expression levels in different cell lines as analyzed by immunoblotting. (**D**) Quantitative analysis of VDAC1 levels in the indicated cell lines. (**E**) A Kaplan–Meier plot depicting patient survival rate with correlation to low/medium (blue line) or high (red line) VDAC1 expression levels. The initial numbers of patients for VDAC1 expression levels that were low/medium was 63 and for high 22, *p* = 0.0001. Data were obtained from: http://ualcan.path.uab.edu/ (accessed on 15 May 2022) [44].

Expression levels of VDAC1 in samples from healthy individuals and patients with malignant mesothelioma were assessed in a tissue microarray slide by immunohistochemistry (IHC) using VDAC1-specific antibodies (Figure 1A,B). Representative immuno-stained sections clearly showed marked increases in VDAC1 expression levels in the tumor tissues, relative to its levels in the healthy tissues. This is similar to what we have found for other types of cancers such as in lung, colon, melanoma, and cervical tissues [31,33,34,35].

The expression of VDAC1 in several cell lines was also analyzed using immunoblotting, with VDAC1-specific antibodies, showing the overexpression of VDAC1 in various cancer cell lines by up to five fold relative to VDAC1 levels in the noncancerous or transformed cells (Mef, HEK-293, and HaCat) (Figure 1C,D).

Moreover, the prognostic value of the VDAC1 expression level is reflected in a survival analysis performed on publicly available gene expression datasets of mesothelioma cancer patients (Figure 1E). A Kaplan–Meier analysis assessing patient survival as a function of the relative to VDAC1 mRNA levels ((high (red) and low/medium (blue)) showed that high levels of VDAC1 were associated with low survival rates.

### 3.2. VDAC1 Silencing Inhibits Cell Proliferation

**Figure 2:**
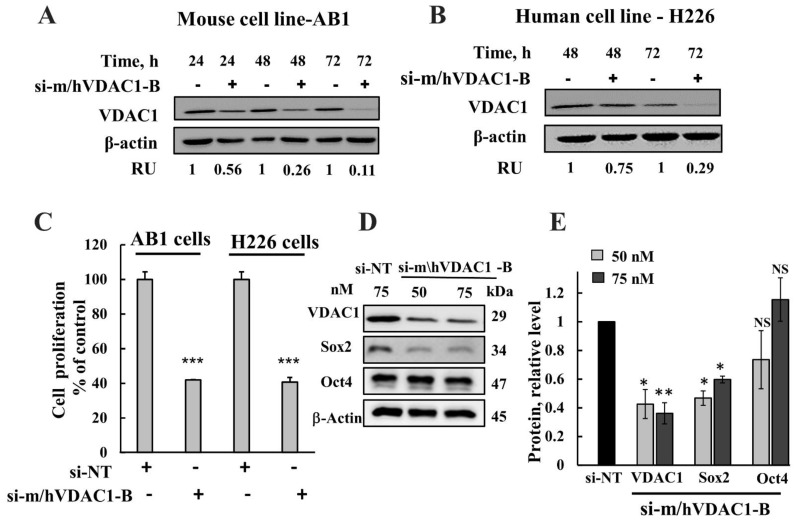
VDAC1 silencing decreases mesothelioma cell proliferation. Mouse (AB1) (**A**,**C**) and human (H226) (**B**,**C**) mesothelioma cancer cell lines were treated with si-NT or si-m/hVDAC1-B (50 nM) for the indicated time, as described in the Methods section. Cells (10 µg) were subjected to immunoblotting using VDAC1 antibodies, and VDAC1 expression levels are quantified and expressed as relative units (RUs) at the bottom of the blot. (**C**) AB1 and H226 cells were transfected with si-NT or si-VDAC1-A (50 nM), and cell proliferation was assayed 48 h post-transfection using the SRB method. (**D**,**E**) H226 cells were transfected with si-NT or si-m/hVDAC1-B (50 nM or 75). The expression of Sox2 and Oct-4 was analyzed by immunoblotting using specific antibodies (**D**), and their expression levels were quantified (**E**). Results are the means ± SEM (n = 3) * *p* < 0.05; ** *p* ≤ 0.01; *** *p* < 0.001; NS-non significant.

Because VDAC1 overexpression in mesothelioma indicates its importance for cancer development and survival, its silencing by means of RNA interference is expected to inhibit cancer cell proliferation and, therefore, tumor growth. In this study, si-RNA recognizing both human and mouse VDAC1 (modified to include 2-*O*-methyl, si-m\hVDAC1-B) was designed and used. As expected, si-m\hVDAC1-B silenced VDAC1 expression in both human mesothelioma H226 and in mouse mesothelioma AB1 cancer cell lines, as analyzed by immunoblotting (Figure 2A,B). The expression of VDAC1 decreased in both cell lines by si-m\hVDAC1-B treatment in a time-dependent manner with about a 70% and 90% decrease observed 72 h post-transfection for H226 and AB1 cells, respectively. It should be noted that the decrease in VDAC1 level upon siRNA treatment is also a function of its degradation rate being different in different cancer cell lines.

The effect of VDAC1 silencing on cell proliferation was analyzed using a sulforhodamine B (SRB) cell viability assay, showing that si-m/hVDAC1-B significantly inhibited cell proliferation in both human and mouse cells (Figure 2C).

Next, we tested the effect of VDAC1 silencing on the expression of cancer stem cells (CSCs), a small subset of cells with the ability to self-renew and maintain tumor growth and recurrence after therapeutic intervention [45]. The percentage of CSCs in the different cell lines varied from 1 to 30%, as judged by their capacity to form neurospheres [46]. CSCs in malignant mesothelioma represent cell subpopulations that can be identified by the expression of specific markers such as Oct4 and SOX2 [47,48]. Thus, the effect of VDAC1 silencing on the expression of Sox2 and Oct4 was analyzed (Figure 2D,E). The results show that Sox2 was significantly decreased (40–60%), while the Oct4 expression level was not significantly changed upon VDAC1 depletion.

### 3.3. si-m/hVDAC1-B Inhibited Tumor Growth in Mesothelioma Xenografts and in a Syngeneic Mice Model

**Figure 3:**
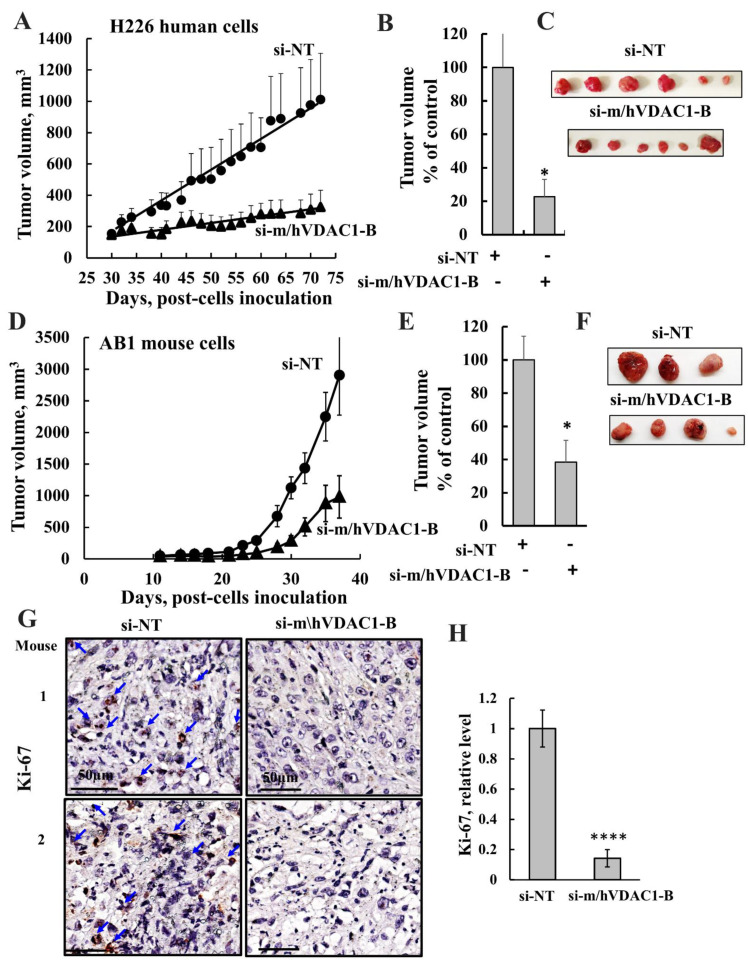
VDAC1 silencing inhibits mesothelioma tumor growth. (**A**,**B**) H226 cells (5 × 10^6^ cells/mouse) were inoculated subcutaneously into nude mice. Tumor volumes were monitored, and on day 30, mice were divided into two groups (n = 6) with similar average tumor volumes (60–110 mm^3^). Xenografts were injected every 3 days with si-NT (100 nM) or si-m/hVDAC1-B (100 nM). The sizes of the tumors were measured, and average tumor volumes were calculated using as described in the Method section and presented as means ± SEM. (**B**) Tumor growth from mouse H226 cell xenografts at day 72 presented as % of control. (**C**) Tumor photography. Results are the means ± SEM (n = 6), * *p* ≤ 0.05. (**D**,**E**) AB1 cells (5 × 10^5^ cells/mouse) were subcutaneously inoculated into BALB/c mice. Tumor volumes were monitored, and on day 12, mice were divided into two groups (n = 6) with similar average tumor volumes (60–100 mm^3^). The tumors were injected three times a week with si-NT or si-m/hVDAC1-B treatment to a final concentration of 100 nM. The sizes of the xenografts were measured, and average tumor volumes were calculated and are presented as means ± SEM. (**E**) Tumor growth from mouse AB1 cell xenografts at day 36. (**F**) Tumors photography. Results are the means ± SEM (n = 6), * *p* ≤ 0.05. (**G**,**H**) Representative Ki-67 IF stained sections from si-NT-treated and si-m/hVDAC1-B-treated H226-derived tumors, **** *p* ≤ 0.0001.

The effects of silencing VDAC1 expression using si-m/hVDAC1-B on mesothelioma tumors were analyzed on established subcutaneous models of mesothelioma using human cells, NCI-H226, and an allogeneic mouse model using AB1 mouse cells. Tumors were treated intratumorally every three days with si-NT or si-m/hVDAC1-B to a final concentration of 100 nM, and tumor volume was followed for 72- and 36-days post cell inoculation for H226 and AB1 cells, respectively. For H226-derived tumors, in the si-NT group, the tumor volume increased from an average of ~150 mm^3^ to 1010 mm^3^, while in si-m/hVDAC1-B-treated tumors, the volume increased from ~150 mm^3^ to 324 mm^3^—an inhibition of 80% in tumor growth (Figure 3A,B).

The effects of si-m/hVDAC1-B on tumor growth were also tested in a syngeneic model using mouse AB1 cells and Blab/c mice. Here, tumors (60–150 mm^3^) were treated with si-NT or si-m/hVDAC1-B injected to a final concentration of 100 nM. The rate of tumor growth in this syngeneic model was faster than in the H226 xerographs, reaching over 3000 mm^3^ in 36 days compared to H226-derived tumors, which reached 1000 mm^3^ in 72 days (Figure 3A,D). The si-m/hVDAC1-B inhibition of tumor growth relative to the si-NT-treated group was only 60% (Figure 3B,E).

Analysis of the staining intensity of proliferation marker Ki-67 in si-NT and si-m/hVDAC1-B-treated tumors indicated a decrease of about 85% in intensity (Figure 3G,H), suggesting that proliferation is inhibited in the tumors with reduced VDAC1 levels.

### 3.4. si-m/hVDAC1-B Treatment Induced Metabolic Reprogramming in a Tumor Xenograft Mouse Model

**Figure 4:**
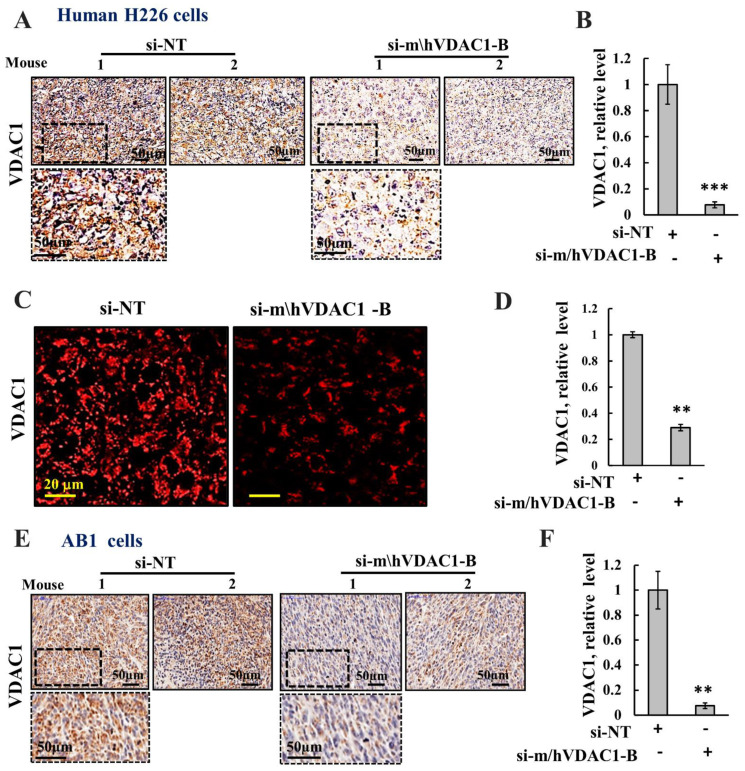
si-m/hVDAC1-B downregulates VDAC1 expression. (**A**–**D**) Representative VDAC1 IHC (**A**,**B**) and IF (**C**,**D**) stained sections from si-m/hVDAC1-B-treated or si-NT-treated H226-derived tumors and their quantification (**B**,**D**). (**E**,**F**) AB1 cell-derived tumors IHC stained for VDAC1 (**E**) and their quantification (**F**). Results are presented as means ± SEM, ** *p* ≤ 0.01; *** *p* ≤ 0.001.

VDAC1 expression levels in the H226 cell-derived xenograft and AB1 cell-derived syngeneic tumors were analyzed on fixed paraffin-embedded sections using IHC and IF staining and anti-VDAC1 specific antibodies (Figure 4), and the expression levels were quantified (Figure 4B,D,F). The si-m/hVDAC1-B-treated tumor sections showed a decrease of about 90% in the VDAC1-staining intensity in comparison to that of the si-NT-treated tumors in both the xenograft and syngeneic model.

**Figure 5:**
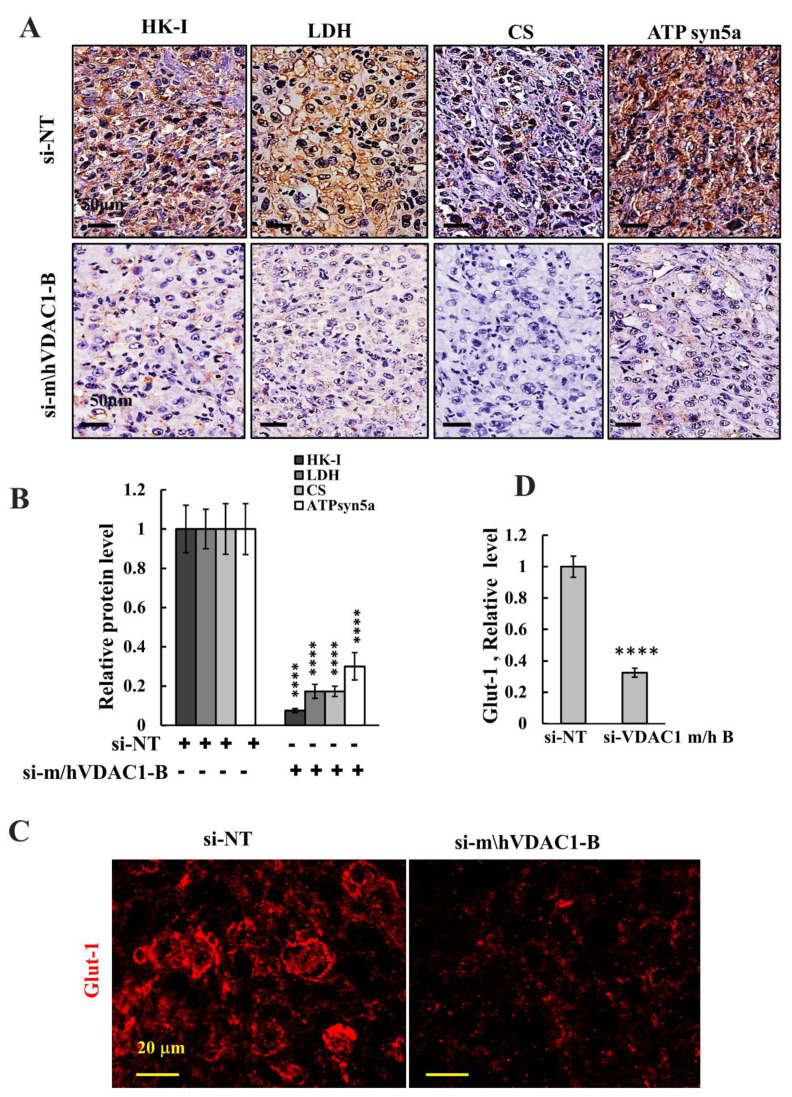
si-m/hVDAC1-B reduces the expression of metabolism-related enzymes. (**A**,**B**) Representative sections from si-m/hVDAC1B-treated or si-NT-treated H226-cell-derived tumors were IHC stained for HK-I, LDH, CS, and ATPsyn5a (**A**) and their quantification (**B**)**.** Tumor sections were also IF stained for Glut-1 (**C**) and its quantification (**D**). Results are the means ± SEM, (n = 3) **** *p* ≤ 0.0001.

Next, we assessed the expression levels of several metabolism-related enzymes in tumor sections from H226 cell-derived tumor that were treated with si-NT or sim/hVDAC1-B and were IHC stained using specific antibodies for glycolytic enzymes, hexokinase 1 (HK-I), lactate dehydrogenase (LDH), the TCA cycle enzyme, citrate synthase (CS), and ATP synthesizing enzyme subunit 5A (Figure 5A,B). In addition, sections were IF stained for the glucose transporter (Glut-1) (Figure 5C,D). The levels of these proteins were highly reduced in the si-m/hVDAC1-B-treated tumors, relative to their levels in si-NT-treated tumors.

The results are consistent with tumor depletion of VDAC1, altering cell metabolism, including mitochondrial and glycolysis activities, in agreement with the concept that cancer cells use a combination of glycolysis and OXPHOS [49,50].

### 3.5. VDAC1 Silencing Modulates the Tumor Microenvironment and Inflammation

**Figure 6:**
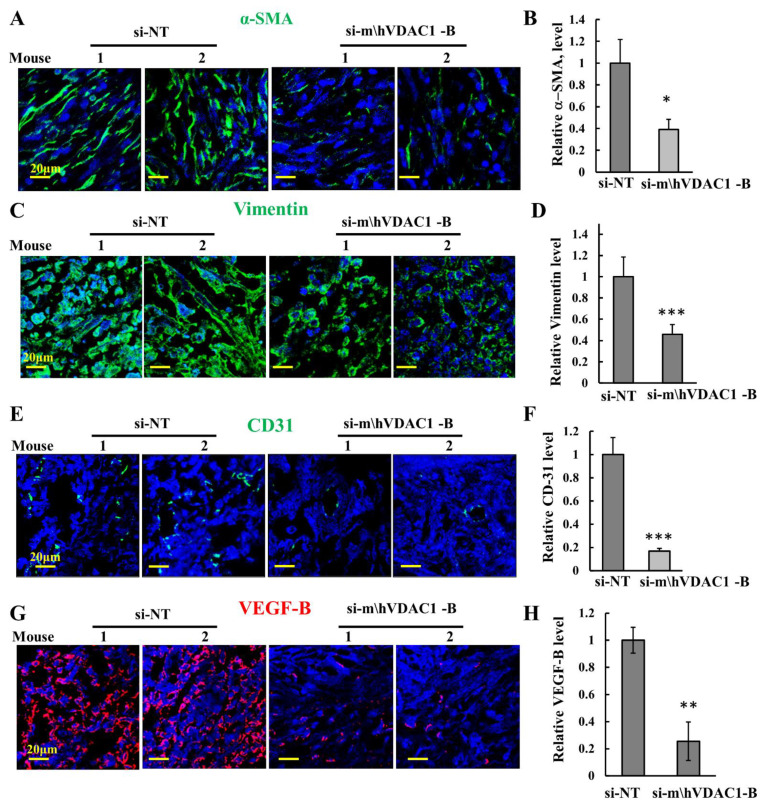
Silencing VDAC1 expression modulates the tumor microenvironment. Representative immunofluorescence images of sections from si-m/hVDAC1-B-treated or si-NT-treated H226-cell-derived tumors stained and quantified for α-SMA (**A**,**B**), and vimentin (**C**,**D**), CD-31 (**E**,**F**), and VEGF (**G**,**H**). Results represent the means ± SEM (n = 3) * *p* ≤ 0.05, ** *p* ≤ 0.01, *** *p* ≤ 0.001.

Tumors are composed of cancer cells and components of the tumor microenvironment (TME) [51]. Alpha-smooth-muscle actin (α-SMA) is expressed in cancer-associated fibroblasts (CAFs), promoting tumor growth and progression [52]. IF-stained for α-SMA in si-NT-treated H226 cell-derived tumors showed staining of a spindle-shape morphology, as expected for CAFs, that was highly decreased in si-m/hVDAC1-B-treated tumors (Figure 6A,B).

Similarly, the strong expression of the fibroblastic marker, vimentin, was reduced in the si-m/hVDAC1-B-treated tumors (Figure 6C,D).

**Figure 7:**
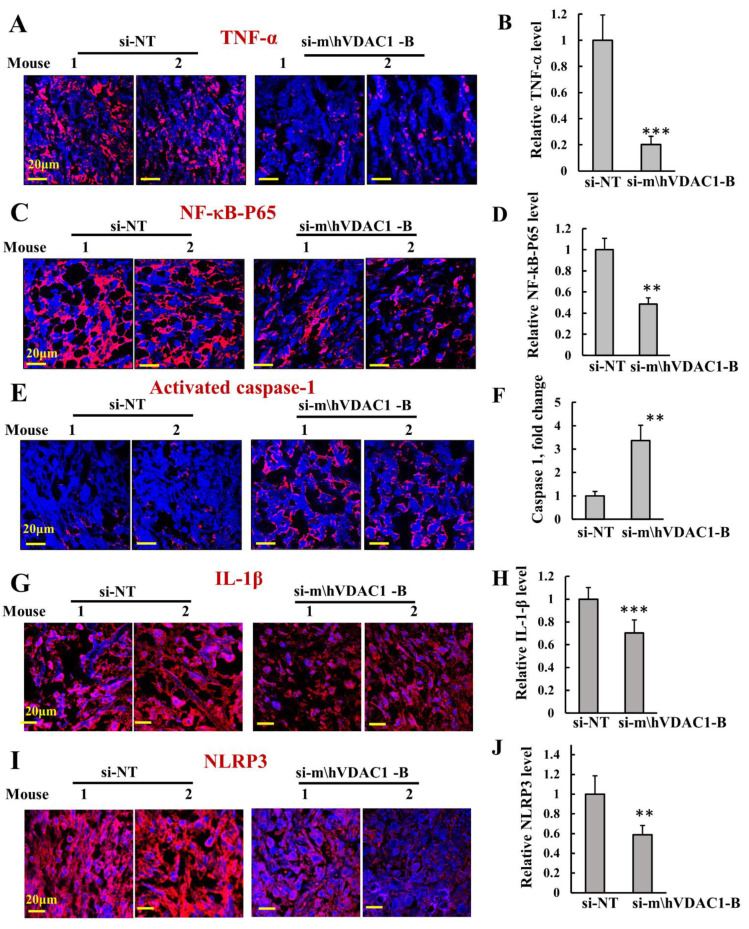
Silencing VDAC1 expression in the tumors altered inflammation. Representative immunofluorescence images of sections from si-m/hVDAC1-B-treated or si-NT-treated H226-cell-derived tumors stained and quantified for TNF-α (**A**,**B**), NF-kB-p65 (**C**,**D**), cleaved caspase-1 (**E**,**F**), IL-1β (**G**,**H**), and NRLP3 (**I**,**J**). Results represent the means ± SEM (n = 3) ** *p* ≤ 0.01; *** *p* ≤ 0.001.

We also assessed angiogenesis by analyzing the expression of CD-31, a specific endothelial marker, and of vascular endothelial growth factor (VEGF) (Figure 6E–H). In agreement with a previous report showing that in mesotheliomas CD-31 staining is rare [53], the expression of CD-31 in mesothelioma was very low, yet it was highly reduced in si-m/hVDAC1-B-treated tumors (Figure 6E,F). VEGF staining was reduced in the si-m/hVDAC1-B-treated tumors (Figure 5G,H). As inflammation plays a critical role in the development and progression of cancer [54], we assessed the effects of si-m/hVDAC1-B tumor treatment on the expression of the pro-inflammatory cytokine, TNF-α, and the nuclear factor-κB (NF-κB) activated form (p-NF-kB-p65), These were analyzed by IF using specific antibodies (Figure 7A–D). VDAC1 depletion in the tumors decreased the expression of both proteins.

Inflammation also involves activation of pro-capsase-1 via NRLP3 inflammasome assembly, leading to the activation of pro-inflammatory molecules such as IL-1β, IL-18, FGF2, and IL-1β. Therefore, we tested the effects of si-m/hVDAC1-B tumor treatment on the expression of NRLP3, activated caspase 1, and IL-1β (Figure 7E–J). si-m/hVDAC1-B-treated tumors showed an over 3-fold increase in activated capsase-1 levels (Figure 7E,F). On the other hand, the levels of IL-1β and NRLP3 were reduced in the si-m/hVDAC1-B-treated tumors (Figure 7G–J). This may suggest that activated caspase-1 is associated with the initiation of inflammatory programmed cell death (pyroptosis) and, in turn, inhibits tumor growth rather than upregulating pro-inflammatory molecules such as IL-1β and IL-18 [55].

### 3.6. si-m/hVDAC1-B Reduced Stemness and Induced Differentiation in Mesothelioma H226-Derived Tumors

CSC subpopulations in malignant mesothelioma were identified by the expression of the transcription factors Sox2 and Oct4, working together to regulate genes required for the self-renewal and pluripotency of embryonic stem cells (ESCs) [48].

**Figure 8:**
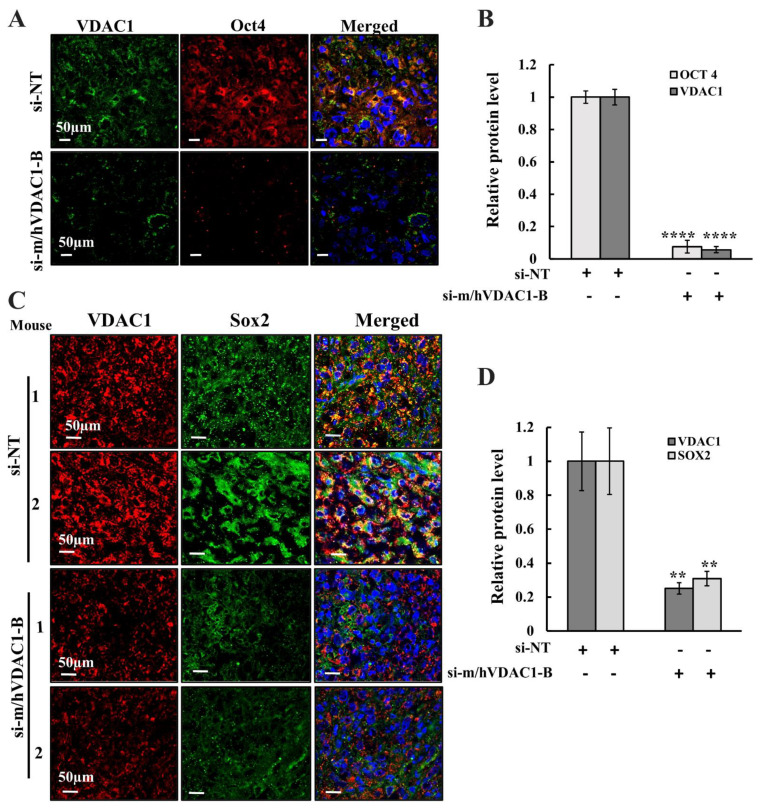
VDAC1 expression depletion in the tumors inhibited stemness. Representative immunofluorescence images of xenograft tumors co-immunostained for VDAC1 and Oct4 (**A**) and for VDAC1 and Sox2 (**C**), and staining intensity was quantified (**B**,**D**). The results are the means ± SEM, ** *p* ≤ 0.01; **** *p* ≤ 0.0001.

Sox2 and Oct4 expression levels analyzed in si-m/hVDAC1-B- and si-NT-treated xenograft tumors showed a drastic decrease in the expression levels of Oct4 (Figure 8A,B) and of Sox2 (Figure 8C,D) in si-m/hVDAC1-B-treated tumors as compared to their levels in si-NT-treated xenografts. This implies that the si-m/hVDAC1-B reduced tumor stemness.

**Figure 9:**
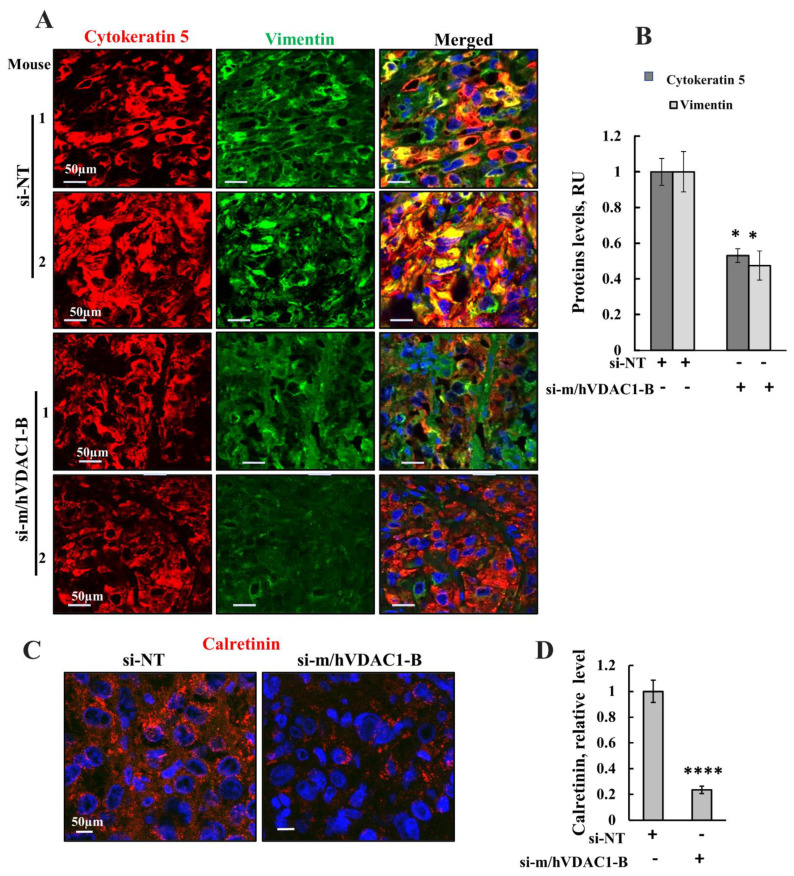
VDAC1 expression depletion decreased the expression of proteins associated with differentiation. Representative immunofluorescence images of sections from H226 cell-derived tumors treated with si-NT or with si-m/hVDAC1-B stained for cytokeratin 5 and vimentin (**A**) and their quantification (**B**), calretinin (**C**), and its quantification (**D**). The results are the means ± SEM, * *p* ≤ 0.05; **** *p* ≤ 0.0001.

Impaired differentiation of cells from adenocarcinoma to pluripotent malignant mesothelial cells is an important hallmark in malignant mesothelioma [56]. To evaluate the impact of VDAC1 silencing on differentiation of mesothelioma xenograft tumor cells, we determined the expression levels of biomarkers for this differentiation vimentin (Figure 9A,B); cytokeratenin5 (Figure 9A,B), a cytoskeletal protein; and calretinin, a calcium-binding protein (Figure 9C,D) [57,58,59] in si-m/hVDAC1-B-treated and si-NT-treated xenograft tumors using IF and specific antibodies (Figure 9). The results showed a reduction in the expression of these differentiation markers, specifically in vimentin and calretinin, and to a lesser extent in cytokeratenin5, in si-m/hVDAC1-B-treated as compared to si-NT-treated tumors (Figure 9). The results suggest that si-m/hVDAC1-B treatment induces differentiation of pluripotent malignant mesothelioma cells, thus reducing the malignancy of tumor cells.

## 4. Discussion

This study demonstrates the potential of silencing the expression of the mitochondrial gatekeeper VDAC1 by specific siRNA to treat mesothelioma; si-m/hVDAC1-B can identify both human and mouse VDAC1 and is modified in several nucleotides to increase stability and decrease immunogenicity [60]. si-m/hVDAC1-B inhibited tumor growth in a mouse model of mesothelioma. Furthermore, the results demonstrate that silencing VDAC1 expression leads to metabolic reprogramming of cancer cells, resulting in an altered tumor microenvironment and reduced inflammation, elimination of cancer stem cells, and induced differentiation.

Considering the challenge in siRNA delivery, and that some of the current treatments of mesothelioma are delivered directly to the tumor via injection close to the tumor area, siRNA can be injected directly into the tumor to overcome delivery problems. This makes mesothelioma treatment using siRNA a promising strategy.

### 4.1. Reprogramming of Cancer Cell Metabolism Induced by VDAC1 Expression Silencing

Tumors require changes in the cellular metabolism and bioenergy of cancer cells [32], while their metabolic adaptation provides the tumor with the precursor needed for the biosynthesis of nucleic acids, fatty acids, cholesterol, and porphyrins [61,62]. Mitochondrial metabolism plays a key role in the survival and development of cancer cells [63].

Here, we demonstrated that interfering with mitochondrial function by the downregulation of VDAC1 expression resulted in reprogrammed cell energy, leading to reduced cell function and survival and differentiation. Depletion of VDAC1 decreased the expression levels of OXPHOS enzymes (i.e., CS and ATPsyn5a), as well as of glycolytic enzymes, (i.e., HK-I and LDH) (Figure 4).

Thus, as reported for other cancers [32,37,39], deletion of VDAC1 resulted in metabolism reprogramming in mesothelioma, while targeting global cell metabolism, rather than an individual enzyme or pathway. This reprogramed metabolism led to the inhibition of cell proliferation and of tumor growth and to an altered tumor microenvironment, modulated inflammation, and elimination of stem cells, while inducing cell differentiation to normal-like cells.

### 4.2. Reprogrammed Metabolism Remodulates the Tumor Microenvironment and Inflammation

A tumor contains not only malignant but also non-transformed cells, with their interactions creating the tumor microenvironment (TME). These include various cell populations such as fibroblasts and immune system cells and vasculature and extracellular matrix (ECM) components [64].

We demonstrated that depleting VDAC1 in cancer cells led to metabolic re-programing and tumor regression and to disruption of tumor–host interactions. This was reflected in the reduced expression of angiogenesis markers such as CD-31 and VEGF, as well as of α-SMA produced by CAFs. VEGF is a potent stimulator of endothelial cell growth and stimulates both physiological and pathological angiogenesis [14]. α-SMA contributes to tumor cell migration and invasion, as well as to metastasis and poorer prognosis [65].

Our results suggest that cancer metabolic re-programming via VDAC1 depletion targets both the cancer and host compartment containing the tumor, remodeling the TME.

Inflammation contributes to development and progression of cancer [54,66]. The pro-inflammatory cytokine TNF-α is secreted by inflammatory cells and is involved in inflammation-associated carcinogenesis. TNF-α could be either pro- or anti-tumorigenic, stimulating cancer cell growth, proliferation, invasion, and tumor angiogenesis, or kill cancer cells [67].

Here, we demonstrated that treatment of mesothelioma tumors with si-m/h-VDAC1-B decreased both TNF-α and the activated p-NF-kB. As TNF-a exerts its biological functions by activating distinct signaling pathways such as NF-κB, a major cell survival signal, the decreased expression of both proteins agrees with si-m/h-VDAC1-B treatment, decreasing tumor growth.

Another important player in inflammation is caspase-1, which has been shown to have both tumorigenic and antitumorigenic effects on cancer development and progression, depending on the type of inflammasome and cancer type [68]. Activating caspase-1 can either lead to cell death or tumor growth by upregulating the secretion of pro-inflammatory molecules such as IL-1β, IL-18, and FGF2 [69]. Caspase-1 overexpression was also shown to induce inflammatory programmed cell death (pyroptosis), also known as inflammatory necrosis, thus inhibiting the growth of tumor cells [55]. Similarly, it has been shown that in mesothelioma tumors that exhibit attenuated NLRP3 inflammasome activation, doxorubicin and cisplatin activate the NLRP3/caspase-1 signaling pathway to activate pyroptosis and suppress tumor proliferation [70]. Indeed, in human cancers, caspase-1 is frequently downregulated, and this caspase-1 deficiency may contribute to tumor growth and the progression of tumor cells [71].

Accumulated evidence shows that mitochondria function in inflammation by sensing and integrating various signals and then relaying these signals to the NLRP3 inflammasome [72]. Thus, as VDAC1 governs mitochondrial functions, its downregulation results not only in reprogramming metabolism and inhibiting cell proliferation, but it also affects inflammation.

### 4.3. Reprogrammed Metabolism Eliminates CSCs by Promoting Their Differentiation

CSCs, with their ability to self-regenerate, are considered to be responsible for initiating tumor growth and recurrence after therapeutic interventions and are associated with tumor resistance to anti-cancer therapies [73].

CSCs have multi-lineage differentiation potential and can undergo dynamic and reversible changes, depending on the surrounding microenvironment, in a process defined as a dynamic race [74]. The nature of CSCs allows them to over-adapt to the TME, as seen under hypoxia [75].

Here, we showed that re-wiring the metabolism by VDAC1 depletion in cells in culture and in tumors resulted in the elimination of CSCs, most probably by inducing their differentiation. In H226 cells in culture, the expression levels of the CSC marker [76] Sox2, but not Oct4, were reduced. This may suggest that the expression of Sox2 is faster and more influenced by the cell energy state than that of Oct4. In this respect, we have shown in our previous studies, both in cells in culture [77] and in mouse models [76], that alterations in stemness and the differentiation process require longer times during which the cells are depleted of VDAC1.

In the mouse model of mesothelioma, the H226 cell-derived xenografts, the levels of Sox2 and Oct4 were high in si-NT-treated tumors, but their levels were highly reduced in tumors treated with si-m/hVDAC1-B.

The decrease in CSCs by VDAC1 silencing may result from arresting their proliferation or from their undergoing differentiation. CSCs have the potential to differentiate into multiple types of cells, including lineage-differentiated cells and non-tumorigenic-differentiated cancer cells, upon exposure to specific differentiation signals and treatments.

Previously, we showed that si-RNA-mediated VDAC silencing in glioblastoma leads to reprogrammed metabolism and reversed oncogenic properties, inducing differentiation of CSCs into neuronal-like cells [38,39]. Several markers were found to distinguish epithelioid mesothelioma from adenocarcinoma. These differentiation markers include vimentin, cytokeratin5 (CK5), and a calcium binding protein calretinin [57,58,59].

CK5 is associated with the epithelial differentiation process present in progenitor cells, but it is lost in differentiation to mature luminal or myoepithelial [57]. Similarly, calretinin and vimentin levels were high in adenocarcinoma relative to their levels in epithelioid mesothelioma [59].

si-m/hVDAC1-B-treated tumors showed a significant reduction in the expression of these markers, specifically in vimentin and calretinin levels as compared to their levels in si-NT-treated tumors (Figure 9). These results suggest that si-m/hVDAC1-B treatment induces differentiation of pluripotent malignant mesothelioma cells into terminally differentiated cells, thus reducing the malignancy of tumor cells.

VDAC1 depletion leads to alterations in the expression of several thousands of proteins [39] and affects the metabolism–epigenetics axis of the tumor [38], as the availability of substrates from the mitochondria to the nucleus for chromatin modifications is decreased. The effects of VDAC1 depletion on the interplay between metabolism and epigenetics can explain the multiple effect of its depletion in several cancer hallmarks.

## 5. Conclusions

In summary, here we showed that depletion of VDAC1 in xenografts of mesothelioma cancer altered the expression of key proteins associated with metabolism, cancer stem cells, differentiation, the TME, and inflammation. By downregulating VDAC1, we targeted cell energy and metabolism and other cell functions essential for cancer cell survival. Moreover, we demonstrated that VDAC1 represents an intersection between metabolism and cancer biology, with metabolism reprogramming following VDAC1 silencing not only inhibiting cell proliferation, but also directing the cell toward a differentiated state. Thus, our study shows that VDAC1 depletion represents a trigger for reprogramming malignant cancer cells into a post-mitotic state and probably into terminally differentiated cells, and that this might be a promising therapeutic approach for mesothelioma and various cancers.

## Figures and Tables

**Table 1:** Antibodies used in this study. Antibodies against the indicated protein, their catalogue number, source, and dilutions used in immunoblotting (WB), immunofluorescence (IF), and immunohistochemistry (IHC) experiments are presented below:

Antibody	Source and Cat. No.	Dilution		
		IHC	WB	IF
Rabbit polyclonal anti-VDAC1	Abcam, Cambridge, UK, ab15895	1:400	1:15,000	1:500
Mouse monoclonal anti-VDAC1	Abcam, Cambridge, UK, ab186321	-	-	1:500
Rabbit monoclonal anti-Glut-1	Abcam, Cambridge, UK, ab115730	-	-	1:500
Rabbit monoclonal anti-HK1	Abcam, Cambridge, UK, ab150423	1:100	-	-
Rabbit polyclonal anti-citrate synthetase	Abcam, Cambridge, UK, ab96600	1:500	-	-
Rabbit monoclonal anti-lactate dehydrogenase	Abcam, Cambridge, UK, ab52488	1:2000	-	-
Rabbit polyclonal anti-ATPsyn5a	Abcam, Cambridge, UK, ab151229	1:500	-	-
Rabbit polyclonal anti-Ki-67	Abcam, Cambridge, UK, ab15580	1:250	-	-
Mouse monoclonal anti-SOX2	Abcam, Cambridge, UK, ab171380	-	-	1:200
Rabbit monoclonal anti Oct4	Abcam, Cambridge, UK, ab200834	-	-	1:250
Rabbit monoclonal anti cytokeratin 5	Abcam, Cambridge, UK, ab52635	-	-	1:250
Mouse monoclonal anti-vimentin	Abcam, Cambridge, UK, ab8978	1:200	-	1:200
Rabbit monoclonal anti-calretinin	Abcam, Cambridge, UK, ab92341	1:500	-	1:250
Rabbit polyclonal anti-a-SMA	Abcam, Cambridge, UK, ab5694	-	-	1:500
Rabbit polyclonal anti-CD31	Abcam, Cambridge, UK, ab28364	-	-	1:750
Mouse monoclonal anti-VEGF-B antibody	Santa Cruz Biotechnology, TX (USA), sc-65617	-	-	1:100
Mouse monoclonal anti-TNF-a	Abcam, Cambridge, UK, ab1793	-	-	1:500
Rabbit polyclonal anti-NF-kB p65 (Ser536) antibody	Bioss, MA (USA), BS-092R	-	-	1:250
Goat polyclonal anti-NRLP3	Abcam, Cambridge, UK, ab4207	-	-	1:500
Rabbit polyclonal anti-IL-1β	Abcam, Cambridge, UK, ab9722	-	-	1:500
Donkey anti-mouse-Alexa fluor 488	Abcam, Cambridge, UK, ab150109	-	-	1:750
Goat anti-rabbit IgG-Alexa fluor 555	Abcam, Cambridge, UK, ab150086	-	-	1:850
Goat anti-rabbit Alexa fluor 488	Abcam, Cambridge, UK, ab150078	-	-	1:750
Donkey anti-goat- Alexa fluor 555	Abcam, Cambridge, UK, ab150134	-	-	1:500
Goat anti-mouse-Alexa fluor 555	Abcam, Cambridge, UK, ab150114	-	-	1:750
Goat anti-rabbit HRP	Promega, Wisconsin, W4018	1:1000	1:15,000	-
Donkey anti-mouse HRP	Abcam, Cambridge, UK, ab98799	1:1000	1:15,000	-
Mouse monoclonal anti-β-actin	Millipore, Billerica, MA, MAB1501	-	1:40,000	-
